# Emerging trends and focus of Toll-like receptors in kidney diseases: a 20-year bibliometric analysis

**DOI:** 10.3389/fmed.2025.1599196

**Published:** 2025-06-13

**Authors:** Peihan Liu, Xinyao Luo, Dingyuan Wan, Yupei Li, Baihai Su

**Affiliations:** ^1^Department of Nephrology, Kidney Research Institute, West China Hospital, Sichuan University, Chengdu, China; ^2^Department of Critical Care Medicine, West China Hospital, Sichuan University, Chengdu, China

**Keywords:** kidney diseases, Toll-like receptors, bibliometric analysis, ischemic acute kidney injury, inflammation

## Abstract

**Background:**

An increasing number of studies have explored the role of Toll-like receptors (TLRs) in the pathogenesis of kidney diseases and their corresponding therapeutic potential. However, there is no comprehensive bibliometric analysis in this field. This study aims to investigate the hotspots and evolution of TLRs and kidney disease research over the past two decades.

**Methods:**

Publications from the Web of Science Core Collection database were searched and extracted on December 21, 2024 using the terms “Toll-like receptor” and “kidney disease” (and their synonyms in MeSH). CiteSpace was used to explore publications from January 1, 2000, to December 21, 2024, to visualize the contributions of countries, institutions, journals, and authors, and to detect the evolution of research focus and emerging trends in this field.

**Results:**

A total of 2,505 studies with 101,150 references were included in this study. The United States and China are the leading forces among all countries. The Egyptian Knowledge Bank is the leading institution, and Hans-Joachim Anders is considered the most influential expert in this field. PLOS One is the journal with the most publications, and Journal of Immunology is the most co-cited journal. According to the co-citation analysis, COVID-19 is the latest research hotspot. Additionally, both ischemia-reperfusion injury and diabetic nephropathy have been long-standing research hotspots and still hold significant values. Moreover, the use of TLR inhibitors as a therapeutic strategy for kidney diseases is increasingly emphasized.

**Conclusion:**

Our study demonstrates a growing understanding of the crucial role of TLRs in kidney diseases over the past two decades. Future research should attach more importance to the identification of novel endogenous ligands for TLRs, which will be critical for developing TLR inhibitors as a viable therapeutic strategy.

## 1 Introduction

Approximately 850 million people suffer from kidney diseases, including chronic kidney disease (CKD), acute kidney injury (AKI), and kidney failure, with more than 5 million deaths worldwide annually (1). Kidney diseases are particularly challenging to manage due to their pathological diversity and often asymptomatic nature in early stages. As such, a subset of CKD patients has developed into kidney failure by the time they are diagnosed and require dialysis and kidney transplantation for survival, posing a huge economic burden for global health systems (2). Therefore, emphasis has been placed on identifying effective diagnostic biomarkers and therapeutic targets for kidney diseases, particularly glomerulopathy.

TLRs are a family of pattern-recognition receptors that play vital roles in innate immunity (3). Since the identification of TLR4 as the first identified mammalian TLR member in 1998, the TLR family has expanded to include 10 types in humans (TLR1∼TLR10) and two additional types in mice (TLR1∼TLR9, and TLR11∼13) (3). TLRs can be broadly divided into those localized at the cell membrane surface (TLR1, TLR2, TLR4, TLR5, and TLR6) and those localized in the endoplasmic reticulum, endosomes, and lysosomes (TLR3, TLR7, TLR8, TLR9) (4). TLRs are essentially type I transmembrane glycoproteins with three main structural regions: an extracellular domain containing leucine-rich repeat motifs responsible for specific ligand recognition, a transmembrane domain, and a cytoplasmic Toll/interleukin 1 receptor domain required for downstream signal transduction (5).

**FIGURE 1:**
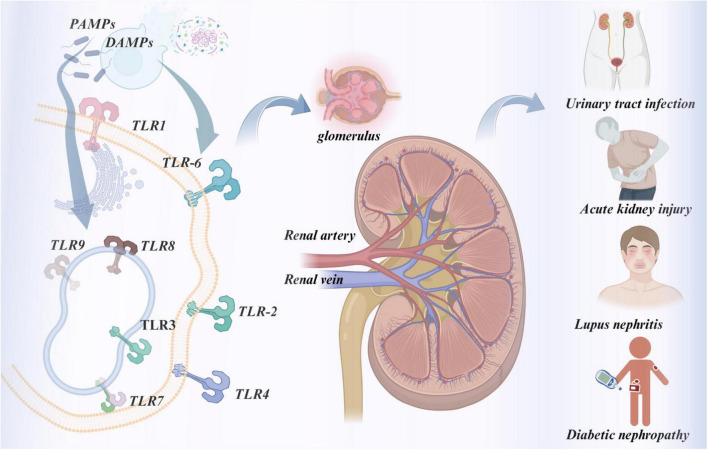
Mechanisms of TLRs in kidney diseases.

TLRs recognize pathogen-associated molecular patterns derived from various classes of pathogens and consequently trigger the innate immune defense against pathogenic infection (6). TLRs not only serve as critical mediators of pathogen recognition in innate immunity, but also participate in the maturation of antigen-presenting cells, thus linking innate and adaptive immune responses (7, 8). In addition, TLRs contribute to non-infectious inflammatory diseases by recognizing endogenous molecules released by damaged tissue, also known as damage-associated molecular patterns (9, 10). Accumulating evidence has revealed that TLRs are involved in the pathogenesis of multiple kidney diseases, including urinary tract infection (11), ischemia-reperfusion injury (12), AKI (13), lupus nephritis (14–16), and diabetic nephropathy (17). The roles of TLRs as potential diagnostic biomarkers and therapeutic targets in kidney diseases are increasingly emphasized, as shown in Figure 1.

Given the important role of TLRs in kidney diseases, recent studies have focused on developing TLR antagonists as potential therapeutic strategies for these conditions. Researchers have investigated the therapeutic effects of drugs (18), natural products (19, 20), or gene modulators (21) on the TLR signaling pathway and explored their potential applications in the treatment of kidney diseases. Moreover, determining the specific TLR isoforms and downstream signaling pathways involved in different kidney diseases can also provide insights into disease heterogeneity and enable the identification of potential biomarkers for disease diagnosis and prognosis (22).

Considering the strong association between TLRs and kidney diseases and their potential clinical value, bibliometric analysis has become a powerful tool for analyzing the knowledge structure and emerging trends in this particular research field (23). To analyze the included papers thoroughly and create visual presentations, specialized software such as CiteSpace, a web-based Java application, is available (24). Through co-citation analysis, CiteSpace quantifies complex information and reveals underlying correlations (24). CiteSpace also identifies research trends and hotspots while objectively analyzing the number of publications produced by various countries, institutes, journals, and authors (24). Given the advances shown in recent studies, there is a dire need to perform a scientific bibliometric analysis of studies linking TLRs and kidney diseases.

Despite the growing attention to the role of TLRs in kidney diseases, no comprehensive bibliometric analysis exists in this field. In this work, we retrieved research data on TLRs and kidney diseases in the Web of Science Core Collection database and used CiteSpace to investigate the contributions of the main countries, institutions, journals, and authors, and to detect current progress and emerging trends in this research field.

## 2 Materials and methods

### 2.1 Search strategy

**FIGURE 2:**
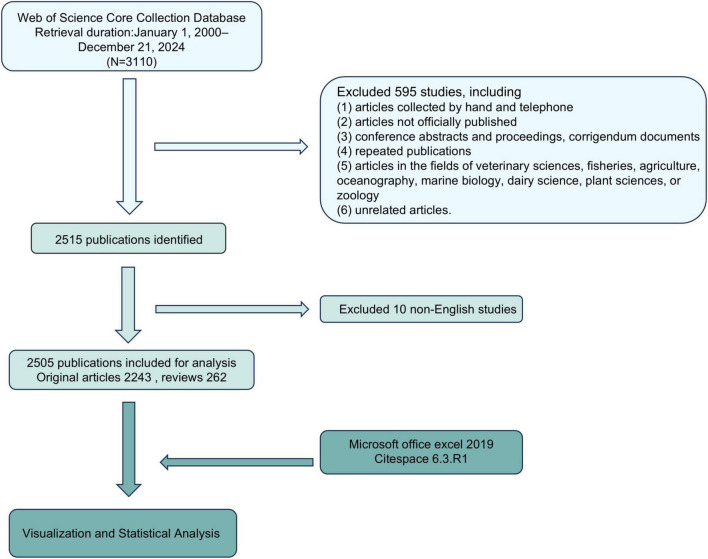
PRISMA flow chart of the study.

According to the PRISMA flow chart (Figure 2), data was retrieved from Clarivate Analytics’s Web of Science Core Collection database, including SCI-EXPANDED, SSCI, AHCI, CPCI-S, CPCI-SSH, ESCI, IC and CCR-EXPANDED. The search terms were as follows: TS = [(TLR OR “Toll-like Receptor”) AND (renal OR kidney OR nephrology OR nephropathy OR nephritis)]. No restrictions were placed on publication types. As the number and annual increment of publications associated with TLRs and kidney diseases before the year 2000 were relatively low, we chose the year 2000 as the starting point for our data retrieval. Additionally, December 21, 2024 was chosen as the endpoint.

The inclusion criteria were as follows: (1) peer-reviewed published original articles on TLRs and kidney diseases, including basic and clinical research; (2) reviews on TLRs and kidney diseases; (3) articles published from January 1, 2000–December 21, 2024; (4) written in English; and (5) articles retrieved from the Web of Science Core Collection database.

The exclusion criteria were as follows: (1) articles collected by hand and telephone; (2) articles not officially published; (3) conference abstracts and proceedings, corrigendum documents; (4) repeated publications; (5) articles in the fields of veterinary sciences, fisheries, agriculture, oceanography, marine biology, dairy science, plant sciences, or zoology and (6) unrelated articles.

### 2.2 Data collection

All data from the Web of Science, including the study title, abstract, author, keywords, countries/regions, institutions, funding agencies, and journals, were downloaded as plain text versions and imported into CiteSpace V6.3. R6, 64-bit for analysis. At the same time, information on the number of annual publications, research types, and journal publications was also collected.

### 2.3 Bibliometric analysis

We summarized the number of articles published by different countries, institutions, journals, and authors. Moreover, the annual number of publications from various countries and institutions was identified via the Web of Science database. The collaborations among countries, institutions, and authors were analyzed through CiteSpace. In terms of parameter settings, the time span was set to 2000–2024, and the time slice was set to one time partition every year. For co-country and co-author analysis, we set the selection criteria as the g-index (*k* = 25), while the selection criteria for co-institution were 10% per slice.

### 2.4 Citation analysis

The data information of “full records and cited references” was imported into CiteSpace. Studies published from January 2000 to December 2024 were chosen with a time slice of 1 year for analysis, and the selection criterion was the g-index (*k* = 25). We selected references as the analysis objects for co-citation analysis and chose cosine as the connection strength. To obtain references that attracted close attention in a period, we further used a burst detection algorithm for analysis.

To find the research topic and characterize the nature of these clusters, we used CiteSpace to extract the key noun phrases from titles, keywords, and abstracts of the citing literature based on three special algorithms: latent semantic indexing, log-likelihood ratio, and mutual information. We chose log-likelihood ratio for the follow-up analysis, as it tends to provide the best results in terms of uniqueness and coverage of topics associated with the clusters.

## 3 Results

### 3.1 General information

The PRISMA flow chart outlines the literature screening process (Figure 2). An initial search of 3,110 studies from the Web of Science Core Collection database between January 1, 2000 and December 21, 2024 was conducted on December 21, 2024. Of these, 595 were excluded due to non-peer-reviewed sources (e.g., conference abstracts) or irrelevant fields (e.g., veterinary medicine). Subsequently, 10 non-English studies were removed, leaving 2,515 publications. After further review, 10 studies were removed due to incomplete metadata or irrelevant nature, and 2,505 studies were finally included for analysis, including 2,243 original articles and 262 reviews, with a total of 101,150 references. These articles were exported to Microsoft Office Excel 2019 and CiteSpace 6.3.R1 for visualization and bibliometric analysis.

The earliest article in the field of TLRs and kidney diseases was published in 1999 by Heine et al. (25), who transfected Chinese hamster ovary TLR2 cDNA into human epithelial kidney 293 cell to investigate whether the expression of TLR2 was essential for endotoxin-induced signal transduction. In the next two decades, the number of publications in this field increased exponentially, surging from 1 to 2,505.

### 3.2 Contribution of countries

**FIGURE 3:**
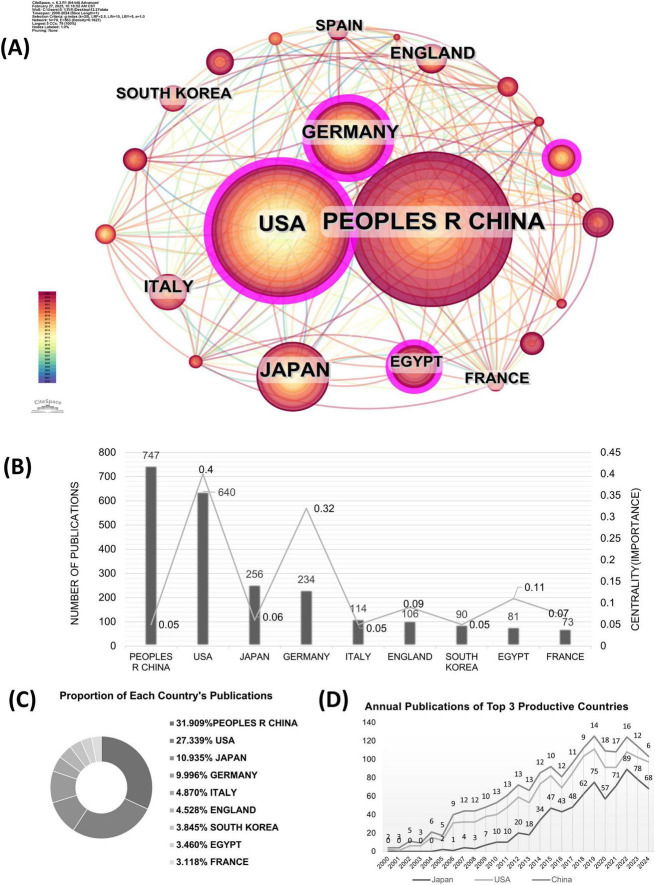
The co-country analysis in the field of TLRs and kidney diseases during January 2000–December 2024. **(A)** The collaboration network of countries. **(B)** Total distribution comparison of the top 9 most published countries and centralities. **(C)** Total publications and centralities comparison of the top 9 most published countries. **(D)** Annual publications of the top three productive countries.

The Co-country analysis results for TLRs and kidney diseases are displayed in Figure 3A, where the circle size represents the number of papers published by each country. The shorter the distance between two circles, the greater the cooperation between two countries. The color of the lines that appear together between countries indicates a chronological order, where cold colors represent earlier cooperation, while warm colors represent cooperation in more recent years. Purple rings indicate that these countries have high centrality (more than 0.1), which means that the node has great influence. Apparently, the most significant node is the United States, which has the second largest number of works and the earliest exploration in this field. The cooperation density was 0.1793, suggesting that cooperation among these countries tends to be strong and stable.

The publication numbers and centrality of the top 9 productive countries are further shown in Figure 3B. The United States and Germany show high centrality values of 0.40 and 0.32, respectively. At the same time, China has 747 publications, followed by the United States with 640, Japan with 256, and Germany 234 with publications. The following countries are Italy, England, South Korea, Egypt, and France, of which the number of publications is relatively lower. Figure 3C shows the proportion of publications from the top 9 countries in this field. The number of publications from the top 9 productive countries accounts for nearly 70% of the total publications, indicating a high degree of national concentration. Figure 3D presents the number of annual publications in the top three productive countries, sorted as China, the United States, and Japan. The number of annual publications in the United States and Japan grew steadily in the first decade, while China’s increase was slightly delayed. From 2015 to 2024, China saw a rapid increase in annual publications and maintained its dominance, while the United States and Japan continued to see modest increases.

### 3.3 Contribution of institutions

**FIGURE 4:**
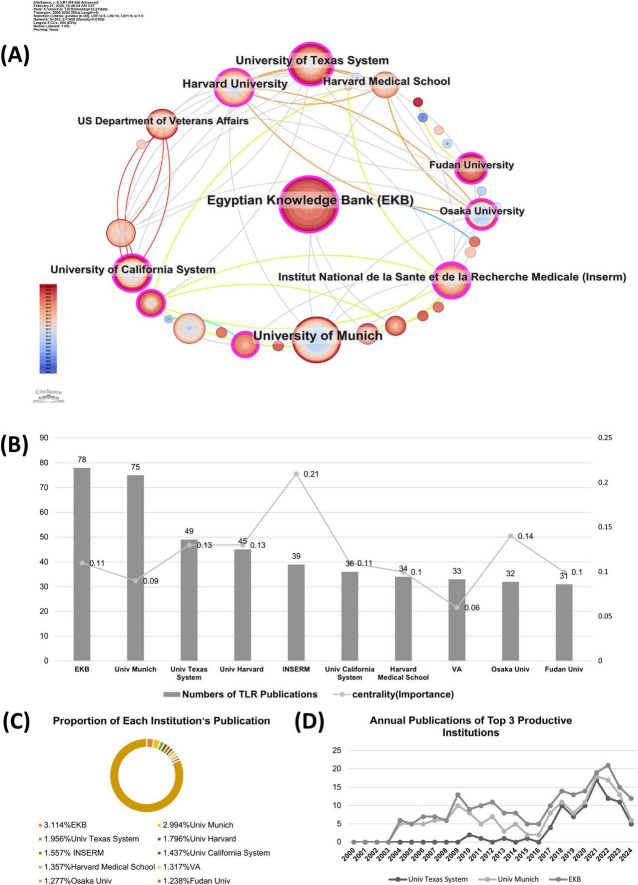
The co-institution analysis in the field of TLRs and kidney diseases during January 2000–December 2024. **(A)** The collaboration network of institutions. **(B)** Total publications and centralities comparison of the top 10 most published institution. **(C)** Proportion of the top 10 most published institutions. **(D)** Annual publications of the top three productive institutions.

Institute cooperation was defined by the presence of two authors’ institutes in the same article. CiteSpace software primarily assesses cooperation among institutes based on the co-occurrence frequency matrix. The results of the co-institute analysis are presented in Figure 4A. The size of an institute’s name represents the number of papers published by that institute. Moreover, a shorter distance between two institutes indicates greater cooperation between them. From the institute distribution map, we can conclude that the research efforts of various institutions are relatively scattered, and the degree of cooperation is relatively low, indicating a need for stronger collaboration. Intuitively, the Egyptian Knowledge Bank is the most significant node in recent years. The University of Texas System and Harvard University in the United States have a close relationship with the US Department of Veterans Affairs, while the Veterans Health Administration in the United States tends to have a relatively independent relationship with other institutions.

Figure 4B further shows the publication numbers and centrality of the top ten institutes by publication volume. The cutting-edge research is concentrated at Egyptian Knowledge Bank (78), University of Texas System (47), Fudan University (31), and Sun Yat-sen University (24). The centrality analyses show that Institut National de la Santé et de la Recherche Médicale has the highest centrality score (centrality = 0.21), indicating that their works are of great significance and originality in this field. Figure 4C presents the proportion of the overall publications by the top ten productive institutes. Research works from these institutions mentioned above account for nearly 18% of the total publications, suggesting that the research of these institutes is relatively scattered. The annual publications from the top 3 active institutions are shown in Figure 4D. In the early decade (2000–2010), the University of Munich has played a leading role in this field, which has been taken over by the Egyptian Knowledge Bank recently from January 2017 to December 2024.

### 3.4 Contribution of scholars

**FIGURE 5:**
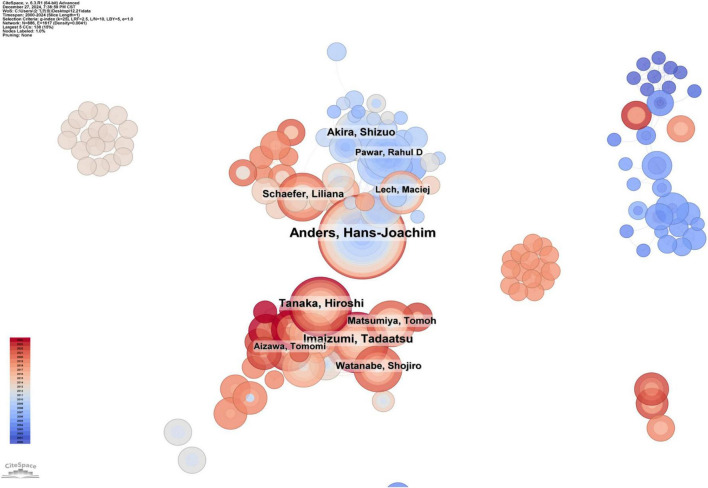
The collaboration network of scholars.

The analysis of co-authors shows the collaborative network of authors in this research field. A co-author distribution map generated by CiteSpace is shown in Figure 5, where the node size represents the number of publications by each author. A shorter distance between nodes indicates greater cooperative activity among authors. The cooperation intensity among these authors was not as strong as the collaboration observed between countries.

The top ten productive authors in this field and their representative research works are summarized in Table 1. Hans-Joachim Anders is the most productive author, with 50 published papers. The research of the Anders group focuses on the activation and regulation of innate immunity in the kidneys. Tadaatsu Imaizumi ranks second with 30 publications, focusing on the function of TLRs and their role in autoimmune diseases, inflammation, and kidney diseases. Akira Shizuo ranks third with 26 publications, mainly studying the role of TLRs in autoimmune kidney diseases, especially lupus nephritis.

**TABLE 1:** Top 10 productive authors and their representative article.

Rank	Author	DOI number	Publication counts	Representative article in this field
1	Anders, Hans-Joachim	10.1681/ASN.2011111077	50	Histones from dying renal cells aggravate kidney injury via TLR2 and TLR4
2	Imaizumi, Tadaatsu	10.1007/s10157-014-1060-4	30	Toll-like receptor 3 signaling contributes to the expression of a neutrophil chemoattractant, CXCL1 in human mesangial cells
3	Akira, Shizuo	10.1681/ASN.2007090982	26	TLR4 signaling mediates inflammation and tissue injury in nephrotoxicity
4	Lech, Maciej	10.1681/ASN.2006101162	19	Inhibition of Toll-like receptor-7 (TLR-7) or TLR-7 plus TLR-9 attenuates glomerulonephritis and lung injury in experimental lupus
5	Matsumiya, Tomoh	10.1093/ndt/gfq270	19	Retinoic acid-inducible gene-I is induced by double-stranded RNA and regulates the expression of CC chemokine ligand (CCL) 5 in human mesangial cells
6	Matanabe, Shojiro	10.1038/pr.2012.165	18	Glomerular expression of fractalkine is induced by polyinosinic-polycytidylic acid in human mesangial cells: possible involvement of fractalkine after viral infection
7	Sato, Miyuki	10.1681/ASN.2011111077	18	Histones from dying renal cells aggravate kidney injury via TLR2 and TLR4
8	Kawaguchi, Shogo	10.1080/14397595.2019.1682768	17	Endothelial expression of fractalkine (CX3CL1) is induced by Toll-like receptor 3 signaling in cultured human glomerular endothelial cells
9	Yoshida, Hidemi	10.1093/ndt/gfq270	16	Retinoic acid-inducible gene-I is induced by double-stranded RNA and regulates the expression of CC chemokine ligand (CCL) 5 in human mesangial cells
10	Tsuruga, Kazushi	10.1620/tjem.228.17	15	Melanoma differentiation-associated gene 5 regulates the expression of a chemokine CXCL10 in human mesangial cells: implications for chronic inflammatory renal diseases

### 3.5 Contribution of journals

Table 2 further summarizes the top 10 cited journals and prolific journals in the research field of TLRs and kidney diseases with their co-citation counts, publication records, and impact factors. As presented, all the top co-cited journals, except Journal of Immunology and Journal of Biological Chemistry, were categorized as Q1 and had a significantly higher IF (from 2.9 to 50.5). The co-citation count refers to the frequency with which two journals are cited together in the same article. The Journal of Immunology ranks first, with a citation count of 1,522. The second is Journal of The American Society of Nephrology (1,115), followed by Proceedings of the National Academy of Sciences of the United States (1,110), Journal of Clinical Investigation (1,108), Kidney International (1,081), and The Journal of Biological Chemistry (1,065).

**TABLE 2:** Top 10 co-cited and prolific journals.

Rank	Co-cited journal	Co-citation count	IF (2023)	JCR (2023)	Rank	Journal	Record	IF (2023)	JCR (2023)
1	Journal of Immunology	1,522	3.6	Q2	1	Plos One	70	2.9	Q1
2	Journal of the American Society of Nephrology	1,115	10.3	Q1	2	Frontiers in Immunology	62	5.7	Q1
3	Proceedings of the National Academy of Sciences of the United States	1,110	9.4	Q1	3	Journal of Immunology	62	3.6	Q2
4	Journal of Clinical Investigation	1,108	13.3	Q1	4	American Journal of Physiology renal Physiology	55	3.7	Q1
5	Kidney International	1,081	14.8	Q1	5	Kidney International	50	14.8	Q1
6	The Journal of Biological Chemistry	1,065	4	Q2	6	International Journal of Molecular Science	49	4.9	Q1
7	Plos One	1,040	2.9	Q1	7	Journal of the American Society of Nephrology	42	10.3	Q1
8	Nature	869	50.5	Q1	8	Scientific Reports	34	3.8	Q1
9	Journal of Experimental Medicine	845	12.8	Q1	9	Nephrology Dialysis Transplantation	30	4.8	Q1
10	Science	833	44.8	Q1	10	Journal of Biological Chemistry	25	4	Q2

Ten of the most prolific journals were classified as Q1 and Q2. The number of articles published in Plos One (70) was the highest, followed by Frontiers in Immunology (62), Journal of Immunology (62), American Journal of Physiology Renal Physiology (55), Kidney International (50), International Journal of Molecular Sciences (49), and Journal of the American Society of Nephrology (42).

### 3.6 Reference co-citation analysis

**FIGURE 6:**
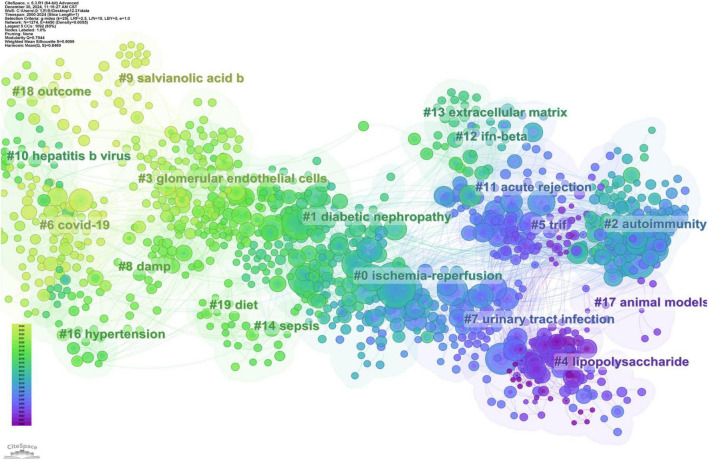
The co-citation knowledge map in the field of TLRs and kidney diseases during January 2000–December 2024. Ten clusters with different research topics were formed, reflecting in different colors on the map. Cluster#6 COVID-19 was the most recent research directions.

CiteSpace integrated networks are synthesized from papers published in 1-year intervals to form an overview of the temporal evolution in TLRs and kidney diseases research. The reference co-citation analysis of TLR and kidney disease research is presented in Figure 6, which generated 19 main co-citation clusters, with 1,274 nodes and 4,450 connections. The modularity Q is 0.7944, and the average silhouette value is 0.9069, implying that the clustering results are highly homogeneous and significant.

The cluster label size is proportional to the number of articles included in the cluster, while the node size refers to the citation number, where the color and thickness of the annual ring indicates the citation frequency among different periods. Line colors correspond directly to the time of citation, meaning that light colors represent more recent years, while the deeper colors represent earlier years.

Based on the LLR algorithm we extracted the noun phrases from the titles, keywords, and abstracts of the citing articles to generate the marks of each cluster. Detailed information on these clusters is summarized in Table 3. According to the reference co-citation cluster marks, ischemic-reperfusion kidney injury represents the latest research hotspot. The specific mechanisms of autoimmunity and diabetic nephropathy have emerged as key research priorities in the field. Furthermore, TLR plays a crucial role in urinary tract infection.

**TABLE 3:** Detailed information of clusters.

Cluster ID	Size	Silhouette	Year	Label (LLR)
0	141	0.83	2008	Ischemia-reperfusion
1	119	0.847	2013	Diabetic nephropathy
2	107	0.887	2006	Autoimmunity
3	101	0.877	2017	Glomerular endothelial cells
4	96	0.952	2000	Lipopolysaccharide
5	86	0.884	2002	TRIF
6	85	0.914	2020	COVID-19
7	59	0.929	2003	Urinary tract infection
8	47	0.914	2015	DAMPs
9	36	0.965	2020	Salvianolic acid B
10	32	0.95	2012	Hepatitis B virus

The most cited reference in each of the clusters indicated the emerging trend of a certain research direction. Coverage reflected the extent to which the ideas, research, or findings of the citing article have been acknowledged and incorporated into subsequent scholarly works, indicating the impact and significance of the citing work within the academic community.

The largest cluster in this field, ischemia-reperfusion (Cluster #0) has 141 members and a mean publication year of 2008. “TLR4 activation mediates kidney ischemia-reperfusion injury” by Wu HL et al. is the reference with the largest citation count of 72 in this cluster (26). Using a mouse model of kidney ischemia-reperfusion injury, they demonstrated that TLR4 signaling was dominant in mediating tubular damage (26). In addition, they also suggested that TLR4 signaling via the adapted protein MyD88 was the dominant pathway contributing to ischemia-reperfusion injury compared with the MyD88-independent pathway (26). The top-ranked item by betweenness centrality (27), not only established the linkage between TLR2 and kidney diseases but also confirmed that TLR2 might participate in ischemia-reperfusion via both MyD88-dependent and MyD88-independent pathways in the pathogenesis of acute ischemic kidney injury (27). These two articles in this cluster could be regarded as the foundation research works in this research field, with far-reaching implications.

The second largest cluster is “diabetic nephropathy” (Cluster #1), consisting of 119 members and with a mean publication year of 2013. “Toll-like receptor 4 promotes tubular inflammation in diabetic nephropathy” is the most cited publication with 48 citations. The work of Lin M et al. demonstrated that the expression of renal tubular TLR4 but not TLR2 was elevated in human diabetic nephropathy biopsies and correlated directly with macrophage infiltration (28). Induced by high glucose, TLR4 could promote proinflammatory effects via IκB/NF-κB activation in human tubular epithelial cell. Furthermore, tubulointerstitial inflammation and IκB/NF-κB activation were also attenuated in TLR4−/− diabetic mice (28). This discovery significantly advances our understanding of the vital role of TLRs in diabetic nephropathy.

“Autoimmunity” (Cluster #2) is the third largest cluster, consisting of 107 members, and has a mean publication year of 2006. “Pathogen recognition and innate immunity” by Hans-Joachim Anders et al. is the reference with the largest citation count of 47 in this cluster (29). This review examined the mechanisms of pathogen recognition in the innate immune system, focusing primarily on host pattern recognition receptors and their associated signaling pathways. “Toll-like receptor 7 and TLR9 dictate autoantibody specificity and have opposing inflammatory and regulatory roles in a murine model of lupus” by SR Christensen is the reference with the second largest citation count of 35 in this cluster. In lupus-prone mice, TLR9 is essential for the generation of DNA autoantibodies, while TLR7 is crucial for generating antibodies to RNA-containing antigens. SR Christensen et al. revealed contrasting functions of TLR9 and TLR7 in lupus pathogenesis: TLR9 exacerbated autoimmune diseases by activating lymphocytes and plasmacytoid dendritic cells, whereas TLR7 served as a protective factor by reducing disease severity and lymphocyte activation (30).

The most recent cluster in the research field is “COVID-19” (Cluster #6), with 85 members and a mean publication year of 2020. The Second-ranked item by citation in this cluster is by Vázquez-Carballo C, with a citation count of 18. This study showed that TLR4 played a key inflammatory regulatory role in the acute phase of AKI and the transition from AKI to CKD. Therefore, anti-inflammatory therapy targeting TLRs, such as TLR4, is considered a potential strategy to reduce kidney injury and improve prognosis.

### 3.7 Emerging trend and research focus

**FIGURE 7:**
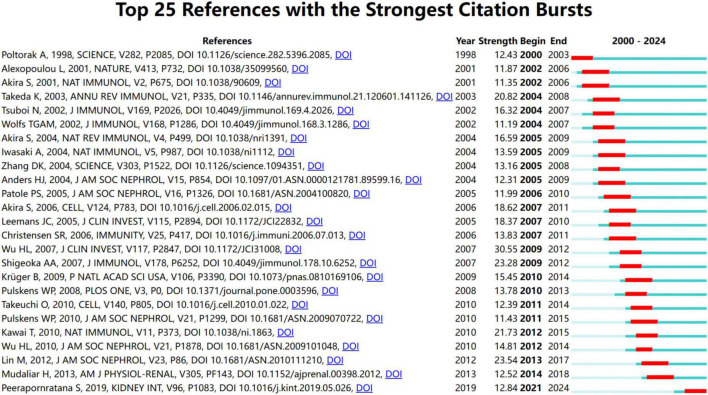
Top 25 references with the strongest citation bursts from January 2000 to December 2024.

The evolution of research focus is highlighted by the citation burst with the measurement of innovative research in this field. A larger burst value indicates a greater innovation of research outcomes, properly representing the hotspots and frontiers in the research field. Figure 7 shows the top 25 references among a total of 244 references with the strongest citation burst from January 2000 to December 2024 and their corresponding beginning and ending year.

The earliest 3 references indicated the emerging trend of research interest from 2000 to 2003. These studies have deepened our understanding of how TLRs function in the recognition of pathogens (31–33). In 1998, Poltorak et al. first revealed that TLR4 in mammals was involved in the response to lipopolysaccharide (32). The median nine references highlighted the emerging research trends that began in 2002 and continued until 2009. These discoveries have focused attention on the mechanism of TLR-mediated immune response and signaling pathways (5, 8, 34–40).

The following references mainly investigated the role of TLRs in the pathogenesis of kidney diseases, especially AKI and diabetic nephropathy (3, 12, 17, 26–29, 41–46). From 2007 to 2014, seven references received great attention, revealing the detailed mechanism by which TLRs contribute to ischemia-reperfusion-induced AKI and sepsis-induced AKI (12, 26, 27, 41, 42, 44, 46). The last 3 references showed the emerging trend from 2013 to 2019, highlighting current hotspots of TLRs and kidney disease research. They mainly pointed out the role of TLRs in mediating tubular inflammation in diabetic nephropathy (17, 28, 45). From these important and insightful references, we could draw a critical map illustrating how TLRs and kidney diseases were associated and TLR-mediated signaling pathways in different kidney diseases.

**FIGURE 8:**
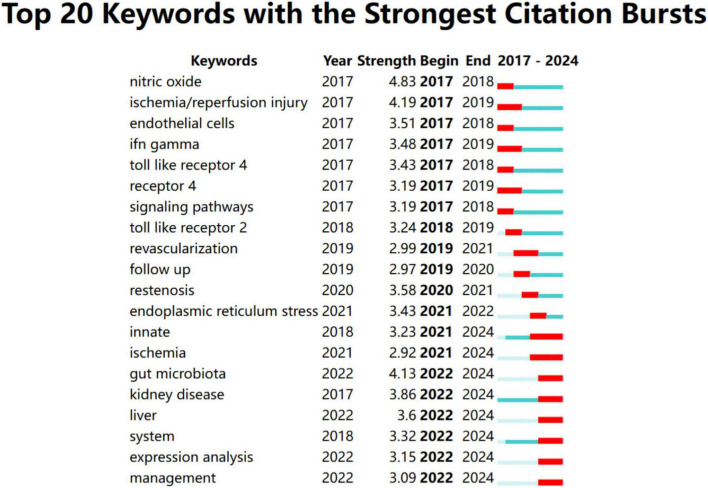
Top 20 keywords with the strongest citation bursts from January 2017 to December 2024.

**FIGURE 9:**
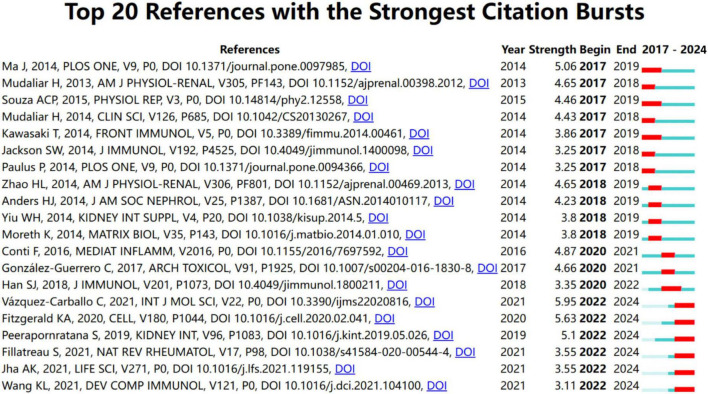
Top 20 references with the strongest citation bursts from January 2017 to December 2024.

To better understand recent research trends in Toll-like receptors (TLRs) and kidney diseases, we analyzed the top 20 keywords and 20 references with the strongest citation bursts starting from 2017, as shown in Figures 8, 9, respectively. The year 2017 was selected as the starting point due to a significant surge in studies on diabetic kidney disease and the role of TLR4 in inflammatory pathways, alongside the rapid emergence of research on novel therapeutic strategies, such as TLR inhibitors, marking a pivotal milestone in the field.

Among the top 20 references with the strongest citation burst from January 2017 to December 2024, the articles authored by Ma J, Mudaliar H, Souza ACP, Kawasaki T, and Ma J were the top five with the strongest burst strength (5.06, 4.65, 4.46, 4.43, and 3.86) (17, 45, 47–49).

Most of them investigated the role of TLR4 in the pathogenesis of diabetic nephropathy and AKI, except for the article by Kawasaki T, which is associated with renal graft ischemia-reperfusion injury (47). The first article by Ma J demonstrated that TLR4 played a key role in the pathogenesis of diabetic nephropathy, and TLR4 activation can lead to podocyte and tubular epithelial cell injury, inflammatory response, and interstitial fibrosis. These findings suggest that the inhibition of TLR4 may be a potential therapeutic target for diabetic nephropathy (45). The last article by Kawasaki T demonstrated TLR regulated immune responses through the activation of NF-κB and IRFs pathways, and thus played a key role in anti-pathogen defense (47). The study by Souza ACP showed TLR4 accelerated, through activation of inflammatory vesicles and dysregulation of the immune response, the progression of renal fibrosis and CKD (49). Mudaliar H also revealed that TLR2 and TLR4 promote inflammation in diabetic nephropathy through activation of NF-κB, and their inhibition may be a new therapeutic target (48). The most recent article in the list, authored by Fillatreau S, had a burst strength of 3.55, summarizing the role of TLR4 in AKI, highlighting that TLR4 exacerbated kidney damage by triggering an inflammatory response, and targeting TLR4 might be a potential strategy for AKI treatment (50). They also highlighted the importance of further research in comprehending the mechanisms underlying S-AKI and facilitating the transfer of knowledge from laboratory experiments, large-scale population studies, and clinical trials (51).

## 4 Discussion

This study conducts the first bibliometric analysis on existing research with keywords “TLR OR ‘Toll-like Receptor’) and (renal OR kidney OR nephrology OR nephropathy OR nephritis” and “kidney disease”, aiming to explore the relevant information in this field. In the past two decades, researchers have paid attention to the role of TLRs as therapeutic targets and diagnostic indicators in kidney diseases. Bibliometric analysis has enabled the assessment of global academic output and the prediction of future research directions regarding TLRs and kidney diseases.

According to the results, most of the contributions to the research come from developed countries, and it is encouraging to see that China, as a developing country, is included in the list as well. Over the past two decades, the United States and China have played a dominant role, contributing to more than half of the publications in this field. Egyptian Knowledge Bank (EKB) contributes greatly to research in this field, focusing on the role of signaling pathways in kidney and liver injury (52–55). Although this field of research in China lagged at first, Wuhan University and Fudan University in China made significant contributions. Their research mostly concentrated on the therapeutic value associated with the TLR signaling pathway (56–63). Germany has made great academic achievements, but the number of published articles is still far behind that in China and the United States. The German University of Munich has an absolute leading position in the field, with the second highest number of publications. Their research mainly focused on the relationship between immune glomerulonephritis and TLRs, especially TLR7 and TLR9 (64–66), and the mechanism by which TLR2 and TLR4 mediate ischemic kidney injury (12, 67).

Hans-Joachim Anders from the University of Munich has become the most influential expert in research and has led the development of TLRs and kidney disease research in recent decades. The most representative article of his group demonstrated that histones from dying renal cells could induce proinflammatory cytokines through TLR2 and TLR4, thus contributing to the pathogenesis of AKI, and could be regarded as milestone research that inspires follow-up studies (67). Tadaatsu Imaizumi and Akira Shizuo have also made great achievements in this research field, and their publications are of great value (41, 68). Imaizumi et al. revealed that TLR3 was involved in the signaling of mesangial CXCL1 expression, a neutrophil chemoattractant cytokine, induced by viral or “pseudoviral” immunity in inflammatory kidney disease (69). Akira Shizuo et al. found that in TLR9-deficient lupus-prone mice, the production of anti-dsDNA and anti-chromatin autoantibodies was specifically suppressed, while the levels of other autoantibodies, such as anti-Smith antibodies, were elevated (70).

A journal is one of the indicators of the value of research. According to our analysis, most of the studies in this field were primarily published in influential journals, such as PLoS One, and those with more co-citation count were primarily published in journals with international renown, such as Journal of the American Society of Nephrology, Journal of Clinical Investigation, Kidney International, Nature, and Science. These results indicated that the field of TLRs and kidney diseases has attracted much attention from top scholars and has been widely recognized for its value.

With a co-citation analysis and burst detection, bibliometric analysis can offer another most important information on the research focus and emerging trends of a certain field. The marks of clusters in reference co-citation analysis revealed that the research focus in this field has been constantly changing over the past two decades: from TIRF (Cluster #5), urinary tract infection (Cluster #3), autoimmunity (Cluster #2) and ischemia-reperfusion (Cluster #0) at the beginning of the twenty-first century, to Hepatitis B virus (Cluster #10), diabetic nephropathy (Cluster #1), DAMPs (Cluster #8), glomerular endothelial cells (Cluster #7), COVID-19 (Cluster #6), and salvianolic acid b (Cluster #9) in the last decade.

According to the co-citation analysis, the role of TLRs in AKI was widely noticed and studied, especially TLR4 and TLR2. One of the basic studies in this direction was published in Journal of Clinical Investigation by Wu HL in 2007, the most cited article in Cluster #0, and one of the references with the strongest citation burst from January 2000 to December 2024 (26). Wu HL et al. found a significant increase in TLR4 expression by tubular epithelial cells and infiltrating leukocytes in an experimental murine model of ischemia-reperfusion injury, and further confirmed that MyD88 was the dominant pathway for TLR4 (26). Moreover, mice deficient in TLR4 were protected from kidney ischemia-reperfusion injury (26). The study by Shigeoka AA revealed that TLR2-deficient mice were better protected from ischemic renal injury than MyD88-deficient mice, indicating that TLR2 functions through both MyD88-dependent and MyD88-independent pathways, while TLR4 function entirely depends on MyD88 (27). Wu et al. further demonstrated that high-mobility group box 1 (HGMB1), a nuclear protein released during cellular injury, stimulated TLR4-mediated ischemia-reperfusion kidney injury (42). Chen et al. showed that TLR4 expression was also observed in endothelial cells, in addition to renal tubular cells. In their study, HGMB1-dependent activation of TLR4 increased adhesion molecule expression by endothelial cells in wild-type mice but not in TLR4-deficient mice (13). In 2021, Zhang et al. demonstrated that the HMGB1-TLR4-IL-23-IL-17A axis played a critical role in regulating inflammation, oxidative stress, apoptosis, and renal injury in ischemia-reperfusion injury by facilitating the recruitment and migration of neutrophils (71). Recently, numerous natural and synthetic compounds have been investigated, which have shown protection against AKI through the inhibition of TLR4 signaling in various pre-clinical AKI models (19, 72–79). To date, therapeutic strategies targeting TLR2 and TLR4 signaling for AKI will still have great research potential and value (17). In addition, current studies have indicated a close association between TLR9 and AKI, with TLR9 activation likely playing a role in AKI development and progression by promoting inflammation, oxidative stress, and disrupting renal immune regulation (80). However, the precise molecular regulatory mechanisms between TLR9 and AKI still need to be further elucidated. TLR4 significantly contributes to AKI by activating the JNK/NF-κB signaling cascade, which drives proinflammatory cytokine production and tubular damage. Studies in ischemia-reperfusion models demonstrate that mindin deficiency mitigates TLR4-driven inflammation, reducing renal injury (81). Similarly, TLR9 exacerbates AKI by sensing mitochondrial DNA released from injured tubular cells, triggering innate immune activation and amplifying systemic inflammation and mortality in ischemia-reperfusion models supplemented with mitochondrial injection (82).

In addition to AKI, diabetic nephropathy has also occupied a place in the field of TLRs and kidney diseases. Inflammation plays a significant role in the development and progression of diabetic nephropathy. Activation of the innate immune system and induction of pro-inflammatory cascade via TLRs, specifically TLR2 and TLR4, has been implicated in the pathogenesis of diabetic nephropathy (48). In the article cited most in Cluster #1, Lin et al. demonstrated an increase in TLR4, but not TLR2, expression in human biopsies with diabetic nephropathy (28). *In vitro*, silencing TLR4 attenuated high glucose-induced NF-κB activation and inhibited the downstream synthesis of pro-inflammatory cytokines (28). Conversely, silencing TLR2 showed no difference in NF-κB activation. Moreover, a decrease in albuminuria, renal dysfunction, NF-κB activation, and interstitial macrophage infiltration were observed in TLR4 −/− diabetic mice, compared with wild-type mice (28). To determine the relative contribution of TLR2 and TLR4 in diabetic nephropathy, Mudaliar et al. further showed that, *in vitro*, both TLR2 and TLR4 could be stimulated by HMGB1 and mediate NF-κB activation, but only TLR2 expression, not TLR4, was sustained for 7 days under high glucose conditions, implying that long-term pro-inflammation merely depended on TLR2 in diabetic nephropathy (17). Similarly, *in vivo* data showed an increase in tubular TLR2, HMGB1, and fibronectin expression, with no increase in TLR4 expression, suggesting that the HMGB1-TLR2-NF-κB pathway might play a dominant role in mediating inflammation in diabetic nephropathy in the long term (17). In the following years, although the number of studies on diabetic nephropathy is relatively small compared with other directions, according to the result that the references associated with diabetic nephropathy are still in the stage of citation burst, it can be predicted that this field will continue to be an active area of research. Various therapeutic approaches targeting TLRs are being explored to alleviate kidney damage in diabetic nephropathy, including TLR antagonists (83–85) and natural compounds with TLR-modulating properties (20, 86–89). Clinical approaches targeting TLRs in diabetic nephropathy include montelukast, which reduces renal inflammation by suppressing HMGB1/TLR4/NF-κB signaling (85). Additionally, berberine ameliorates proteinuria and renal injury by inhibiting TLR4/NF-κB pathways (90). These strategies highlight the therapeutic potential of TLR modulation, with ongoing exploration of antagonists and natural compounds to mitigate diabetic nephropathy progression (91).

Emerging evidence has suggested that TLR4 binds to SARS-CoV-2, triggering immune responses and worsening kidney damage through inflammation, dysfunction, and thrombosis, according to “COVID-19” (Cluster #6). TLR4 binds to SARS-CoV-2 spike proteins, initiates immune defense through recognition of pathogen-associated molecular patterns, and promotes viral clearance to activate immune responses. Animal experiments have shown that COVID-19 mice lacking the TLR3/TLR4 adapter TRIF are more susceptible and have a higher risk of death, suggesting that the TLRs signaling pathway is critical for antiviral resistance. Over-activation of TLRs in COVID-19 infection may lead to NETosis (neutrophil extracellular trap formation, NETs) and inflammatory vesicle release, which in turn leads to cytokine storm and kidney injury (92). TLR4 is highly expressed in renal tubular epithelial cells, especially in proximal and distal tubules, and is closely associated with inflammatory responses in the kidney. TLR4 and its downstream signaling molecules, including CD14, MyD88, and TRAF6, are up-regulated in renal tubular and renal endothelial cells in COVID-19 patients, and are closely associated with acute tubular necrosis and increased kidney injury. In addition, TLR4 activation may also play a role in renal tubular injury through signaling pathways such as NF-κB and STAT3. In an animal model of ischemia-reperfusion injury, it was observed that TLR4 activation was associated with the stimulation of an inflammatory response, which subsequently exacerbated AKI (93). In addition, TLR4 activation may also trigger renal tubular dysfunction by altering renal tubular ion transport functions, such as inhibiting bicarbonate uptake and inhibiting Na^+^/H^+^ exchange activity (94). Following SARS-CoV-2 infection, TLR4 activation on platelets may be associated with thrombotic events (such as myocardial infarction) in COVID-19 patients, and this thrombosis may affect the kidneys through cardio-renal syndrome. In summary, excessive TLR4 activation in COVID-19 patients can trigger NETosis and cause damage to renal tubular epithelial cells, suggesting that TLR4 inhibition could serve as a potential therapeutic target.

While this bibliometric analysis provides a comprehensive overview of research trends in TLRs and kidney diseases, certain limitations should be acknowledged. First, the study exclusively utilized the Web of Science Core Collection database, encompassing indices such as SCI-EXPANDED, SSCI, AHCI, CPCI-S, CPCI-SSH, ESCI, IC, and CCR-EXPANDED. Although this database is widely recognized for its rigorous indexing and compatibility with CiteSpace’s analytical algorithms, it may not capture all relevant literature, particularly articles indexed in other databases such as PubMed or Google Scholar. This constraint could potentially exclude some studies, especially those in non-English languages or published in journals not indexed by Web of Science, which may introduce a selection bias. Second, the reliance on citation-based metrics, such as co-citation and citation burst analyses, introduces the issue of citation lag. Recently published articles, particularly those from 2023 to 2024, may not have accumulated sufficient citations to reflect their true impact or emerging significance by the search date of December 21, 2024. This lag could underestimate the importance of cutting-edge research, such as novel therapeutic strategies targeting TLRs. Despite these limitations, the Web of Science Core Collection provided a robust dataset of 2,505 studies, enabling a thorough analysis of global research trends, and the use of CiteSpace’s advanced algorithms ensured the identification of key hotspots and frontiers with high reliability. Future studies could complement this analysis by incorporating additional databases and employing alternative metrics to mitigate citation lag.

## 5 Conclusion

To our knowledge, this is the first article to use bibliometric analysis to explore the development and research focus on the field of TLRs and kidney diseases. The United States and China are the leading forces in the field of research. Egyptian Knowledge Bank and Hans-Joachim Anders are the core contributors. The role of TLRs in ischemic-refusion injury and diabetic nephropathy has long been a research hotspot and still has great research potential and value. The relationship between COVID-19, TLRs and kidney diseases is a rising area of active investigation that holds great potential for understanding and treating renal disorders. However, future research is necessary to clarify the specific mechanisms by which TLRs function in nephropathy, for example, diabetic nephropathy, lupus nephritis, IgA nephropathy, and hypertensive kidney injury. An important focus of future studies will be the identification of novel endogenous ligands for TLRs, which will be critical for developing TLR inhibitors as a potential therapeutic strategy for kidney diseases.

## Data Availability

The original contributions presented in the study are included in the article/supplementary material, further inquiries can be directed to the corresponding authors.
